# Delayed loss of UBE3A reduces the expression of Angelman syndrome-associated phenotypes

**DOI:** 10.1186/s13229-019-0277-1

**Published:** 2019-05-22

**Authors:** Monica Sonzogni, Johanna Hakonen, Mireia Bernabé Kleijn, Sara Silva-Santos, Matthew C. Judson, Benjamin D. Philpot, Geeske M. van Woerden, Ype Elgersma

**Affiliations:** 1 Department of Neuroscience and the ENCORE Expertise Center for Neurodevelopmental Disorders, Erasmus MC University Medical Center, 3015 CN Rotterdam, The Netherlands; 2 Neuroscience Center, Department of Cell Biology and Physiology, and Carolina Institute for Developmental Disabilities, University of North Carolina, Chapel Hill, NC USA

**Keywords:** Ube3a, Angelman syndrome, Mouse model, Seizure, Autism spectrum disorder, Phenotype

## Abstract

**Background:**

Angelman syndrome (AS) is a severe neurodevelopmental disorder caused by mutations affecting *UBE3A* gene expression. Previous studies in mice revealed distinct critical periods during neurodevelopment in which reactivation of *Ube3a* gene expression can prevent the onset of behavioral deficits. Whether UBE3A is required for brain function throughout life is unknown. Here, we address the importance of maintaining UBE3A expression after normal brain development.

**Findings:**

Using a conditional mouse, we deleted the *Ube3a* gene at three ages spanning brain maturation. We assessed the consequences of *Ube3a* gene deletion by testing the mice in behavioral tasks previously shown to produce robust phenotypes in AS model mice. Early embryonic deletion of *Ube3a* recapitulated all behavioral deficits of AS mice. In contrast, *Ube3a* gene deletion at 3 or 12 weeks of age did not have a significant effect on most behavioral tasks and did not increase seizure sensitivity.

**Conclusions:**

Taken together, these results emphasize that UBE3A critically impacts early brain development, but plays a more limited role in adulthood. Our findings provide important considerations for upcoming clinical trials in which UBE3A gene expression is reactivated and suggest that even transient UBE3A reinstatement during a critical window of early development is likely to prevent most adverse Angelman syndrome phenotypes. However, sustained UBE3A expression into adulthood is probably needed for optimal clinical benefit.

**Electronic supplementary material:**

The online version of this article (10.1186/s13229-019-0277-1) contains supplementary material, which is available to authorized users.

## Introduction

Loss of the maternally inherited *UBE3A* allele results in Angelman syndrome (AS), a severe neurodevelopmental disorder, which is characterized by severe intellectual disability, motor coordination deficits, absence of speech, abnormal EEG, and behavioral deficits [1]. *UBE3A* gene dosage also appears to be critical with respect to autism spectrum disorder (ASD) [2]. Previous studies showed that there is up to 50% ASD comorbidity in AS individuals [3–5], while overdosage of *UBE3A*, due to copy number variation of the 15q11–13 region, is among the highest genetic risk factors for ASD, accounting for up to 0.4% of all cases [6, 7]. Since duplication of the maternal locus is highly associated with pathogenicity [7–10], it is likely that *UBE3A* (the only maternally expressed gene in this locus) is the major effector of ASD outcome. Indeed, duplications of just the *UBE3A* gene, as well as a gain-of-function *UBE3A* point mutation that renders UBE3A enzymatically hyperactive, have been linked to severe forms of ASD [11–14].

One of the most promising approaches for developing a treatment for AS is based on the activation of the epigenetically silenced paternal *UBE3A* gene. Paternal *UBE3A* is silenced in neurons by a long non-coding *UBE3A-ATS* transcript, which can be activated by either using anti-sense oligonucleotides (ASOs) that target the degradation of this transcript or by topoisomerase inhibitors that interfere with the transcription of the *UBE3A-ATS* [15–17]. These *UBE3A* reinstatement approaches are particularly attractive since they restore UBE3A protein levels without risking over-expression.

Using an inducible *Ube3a* mouse model in which gene expression was genetically reinstated at different time points of brain development, we revealed distinct critical windows during brain development in which *Ube3a* needs to be reactivated to achieve an optimal behavioral rescue [18]. This suggests that early therapeutic intervention is needed for *UBE3A* reinstatement therapy to be fully effective. However, these results also pose a new question: to what extent is UBE3A expression required *after* brain development and should treatment be continued after brain development has taken place?

To address this question, we made use of a conditional mouse model for AS that enabled us to delete the *Ube3a* gene at any desired time point. We found that early embryonic deletion of *Ube3a* recapitulated phenotypes that were previously described for AS mice [19]. In contrast, behavioral deficits were mostly absent when the *Ube3a* gene was deleted in young (3 weeks) or fully adult (12 weeks) mice. These results emphasize that most phenotypes observed in AS mice reflect developmental deficits. Continued UBE3A expression beyond the completion of brain development may not be required for normal performance on most behavioral tasks.

## Methods

### Mouse breeding

We made use of the *Ube3a*^*flox*^ (*Ube3a*^*tm1.1Bdph*^, MGI:5882092) mice as previously described [20]. These mice were maintained in the C57BL/6 J (Charles River Laboratories) background by crossing male B6.*Ube3a*^*m+/pflox*^ mice with C57BL/6 J females. For the behavioral experiments, we used mice in the B6129S2F1 background, which were generated as described below. To generate embryonic deletion of *Ube3a*, B6.*Ube3a*^*m+/pflox*^ female mice were crossed with CAG (CMV early enhancer/chicken actin promoter) CRE- expressing male mice [MGI:2176435; Tg (CAG-cre)13Miya, in the manuscript referred to as *Cre*^*embryo*^] in the 129S2/SvPasCrl background (Charles River Laboratories) [21]. This breeding yielded four experimental groups in a B6129SF1 background: WT mice with and without CRE, and Ube3a^mflox/p+^ mice with and without CRE.

To allow temporal control of *Ube3a* deletion at 3 and 12 weeks of age, B6.*Ube3a*^*m+/pflox*^ female mice were crossed with homozygous 129S2*Cre*^*ERT*^ [MGI:2182767; Tg (CAG-CRE/Esr1*)5Amc/J, also referred to as Tg (CAG-CRE-ERT2)] male mice [22]. This breeding yielded two experimental groups in a 129S2B6F1 background: WT mice with the CAGcre/Esr1 allele and Ube3a^mflox/p+^ mice with the CAGcre/Esr1 allele.

For the seizure susceptibility experiments, we used mice in the 129S2/SvPasCrl background. To that end, B6.*Ube3a*^*mflox/p+*^ mice were backcrossed for four to five generations in the 129S2/SvPasCrl background. Female 129S2.*Ube3a*^*m+/pflox*^ mice were crossed with either Tg (CAG-CRE) or *Cre*^*ERT*^ male mice in the congenic 129S2/SvPasCrl background (backcrossed > 20 generations).

### Mouse husbandry

All mice were group-housed in a barrier facility, in cages that were individually ventilated (IVC; 1145 T cages from Techniplast). Mice were genotyped when they were 4–7 days old and re-genotyped at the completion of the experiments. All animals were kept at 22 ± 2 °C with a 12-h dark and light cycle and provided with mouse chow (801727CRM(P) from Special Dietary Service) and water ad libitum*.* During behavioral testing, mice remained group-housed, except during the nest building test and subsequent forced swim test.

### Tamoxifen treatment and randomization

Three or 12-week-old Cre^ERT^;*Ube3a*^*mflox/p+*^ transgenic mice and their wild type littermates (both sexes) received tamoxifen to induce Cre-mediated deletion of the *Ube3a* gene. Tamoxifen (Sigma-Aldrich) was diluted in vegetable (sunflower) oil at a concentration of 20 mg/ml as previously described [18, 22, 23] and as recommended by the Jackson Laboratories [24]. For five consecutive days, each mouse received 0.10 mg tamoxifen per gram body weight daily by intraperitoneal (IP) injection. The control group received daily IP injections of sunflower oil for five consecutive days (vehicle). Injection of either tamoxifen or vehicle was randomly assigned to the mice and the experimenter was blind to genotype.

### Behavioral test battery

All behavioral experiments were performed during the light period of the light/dark cycle. Both male and female mice were used at the ages indicated in the text. Mice were acclimatized to the testing room for 30 min before each behavioral performance. All behavioral testing and scoring were performed by an experimenter blind to genotype. Behavioral tests were precisely performed as previously described [18, 19] and as listed below:

#### Accelerating rotarod

Motor capabilities were tested by placing the mice on the accelerating rotarod (4–40 rpm, in 5 min; model 7650, Ugo Basile Biological Research Apparatus, Varese, Italy). Mice were tested twice per day with a 45–60-min inter-trial interval for five consecutive days (same hour every day). For each day, the average time spent on the rotarod was calculated, or the time until the mouse made three consecutive wrapping/passive rotations on the rotarod (latency in seconds). The maximum duration of a trial was 5 min.

#### Open field test

In this test, which is useful to test locomotor activity and anxiety, mice were individually placed in a brightly lit 110-cm-diameter circular open field (25 lx in the middle of the arena) and allowed to explore the space for 10 min. The total distance moved by each mouse in the open arena was recorded by an infrared camera (Noldus® Wageningen, NL) connected to the EthoVision® software (Noldus® Wageningen, NL), and the final outcome is indicated as distance moved in centimeters.

#### Marble burying test

Open makrolon (polycarbonate) cages (50 × 26 × 18 cm) were provided with 4 cm of bedding material (Lignocel® Hygenic Animal Bedding, JRS). On top of the bedding material, 20 blue glass marbles were placed in an equidistant 5 × 4 grid and the animals were free to access to the marbles for 30 min. Once the time was run out, the mice were gently removed from the cage. The outcome measured is the number of buried marbles, which were scored as buried when covered more than 50% by bedding material.

#### Nest building test

Mice were single housed for a period of 5 to 7 days before the start of the experiment. Successively, the used nesting material was replaced with around 11 g (11 ± 1) of compressed extra-thick blot filter paper (Bio-rad©). The amount of the unused nest material was weighed and noted daily for a consecutive of 5 days, each day at the same hour.

#### Forced swim test

Mice were placed in a cylindrical transparent tank (27 cm high and 18 cm diameter), filled with water (26 ± 1 °C) 15 cm deep for 6 min. The outcome measured is the time in seconds in which the mouse was immobile. The latency of immobility was only assessed during the last 4 min of the test. The mouse was considered to be immobile when it stopped moving, making only movements necessary to keep its head above water.

#### Susceptibility to audiogenic seizures

Mice were placed in makrolon (polycarbonate) cages (50 × 26 × 18 cm) and audiogenic seizures were induced by vigorously scraping scissors across the metal grating of the cage lid (which creates approximately a 100 dB sound). This noise was generated for 20 s, or less if a tonic-clonic seizure developed before that time. Susceptible mice responded with wild running and leaping followed by a tonic-clonic seizure, which typically lasted 10–20 s.

### Western blot analysis and immunohistochemistry

Mice were sacrificed at 20–25 weeks of age, for subsequent analysis. For Western blots analysis, approximately 20 μg of protein lysate were loaded on 4–12% SDS-PAGE gel (Bio-Rad) and transferred on nitrocellulose membranes to be then incubated with anti-UBE3A antibody (E8655 Sigma-Aldrich; 1:1000) and anti-actin antibody (MAB1501R, Millipore; 1: 20000). Briefly, membranes were blocked in 4% TBS milk solution for 1 h at room temperature and incubated at 4 °C overnight, rotating end over end, with the primary antibody dissolved in 2% TBS-T milk solution. The day after membranes were washed three times for 10 min with TBS-T and incubated with the secondary antibody, a fluorophore-conjugated goat anti-mouse antibody (IR Dye 800CW, Westburg; 1:15000), dissolved in 2% TBS-T milk solution for 1 h. At the end of the incubation, membranes were washed three times for 10 min with TBS and the resulting blots were analyzed and quantified using a LI-COR Odyssey Scanner and Odyssey 3.0 software.

For immunohistochemistry, mice were sedated with 0.15 ml Nembutal (60 mg/kg), transcardially perfused and the brains were post-fixed with 4% paraformaldehyde in sodium phosphate buffer (PB) for 2 h. After incubation in 10% sucrose (in 0.1 M phosphate buffer) overnight, brains were embedded in a sucrose/gelatin mixture (10 and 12%, respectively). Brain sections were cut on a microtome (SM2000R; Leica Microsystems, Rijswijk, Netherlands) at a thickness of 40 μm and treated with peroxidase (H_2_O_2_). The brain sections were then washed in PBS and were incubated for 1 h in blocking buffer containing 10% horse serum, 0.5% Triton X-100 in PBS. Subsequently, sections were incubated for 48–72 h in 2% normal horse serum, 0.5% Triton X-100 incubation buffer in PBS with primary antibody (mouse anti-E6AP, clone 3E5 Sigma–Aldrich; 1:750). The secondary antibody (anti-mouse HRP, P0447 Dako; 1:200) was detected by 3,3-diaminobenzidine (DAB) as the chromogen, and DAB sections were analyzed and photographed using a Nanozoomer scanner.

### Statistics

All data were statistically analyzed using IBM SPSS software, and *P* values less than 0.05 were considered significant. Statistical analysis was performed using univariate ANOVA (Kruskal-Wallis statistic test when data were non-normally distributed) or two way-repeated measures ANOVA with Bonferroni’s and Dunnet/Mann-Whitney *U* test post hoc comparison (see the Additional files for more details).

## Results

**Fig. 1:**
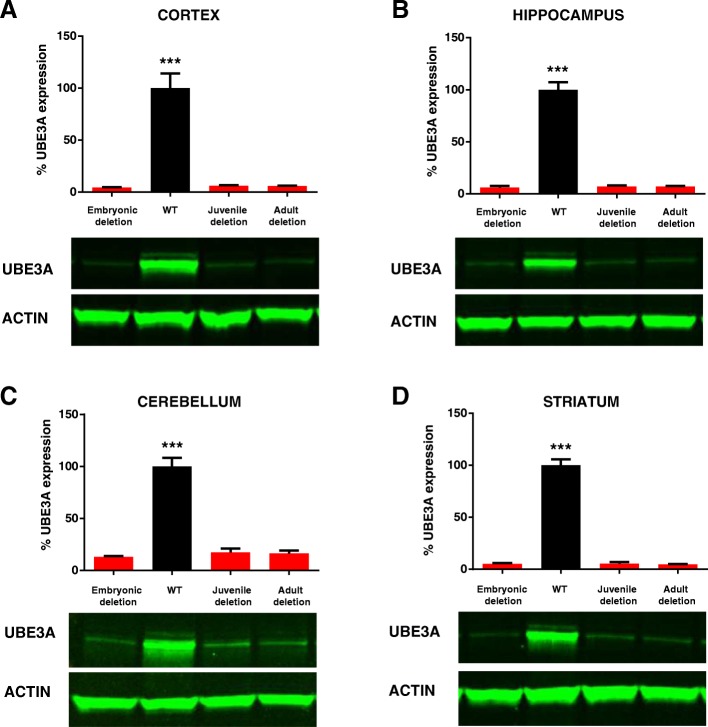
Loss of UBE3A expression upon juvenile and adult *Ube3a* gene deletion. UBE3A Western blot analysis of wild-type mice and mice in which the *Ube3a* gene deletion is induced at 3 weeks (‘juvenile deletion’) or at 12 weeks ‘adult deletion’. Mice were sacrificed between 22 and 25 weeks of age. The analysis shows that loss of UBE3A expression in the cortex (**a**), hippocampus (**b**), cerebellum (**c**) and striatum (**d**) of these mice is comparable to mice in which the *Ube3a* gene is absent throughout development (‘embryonic deletion’) (*N* = 3 per genotype). Data shown are means with SEM (see methods and Additional file 1 for statistical tests)

**Fig. 2:**
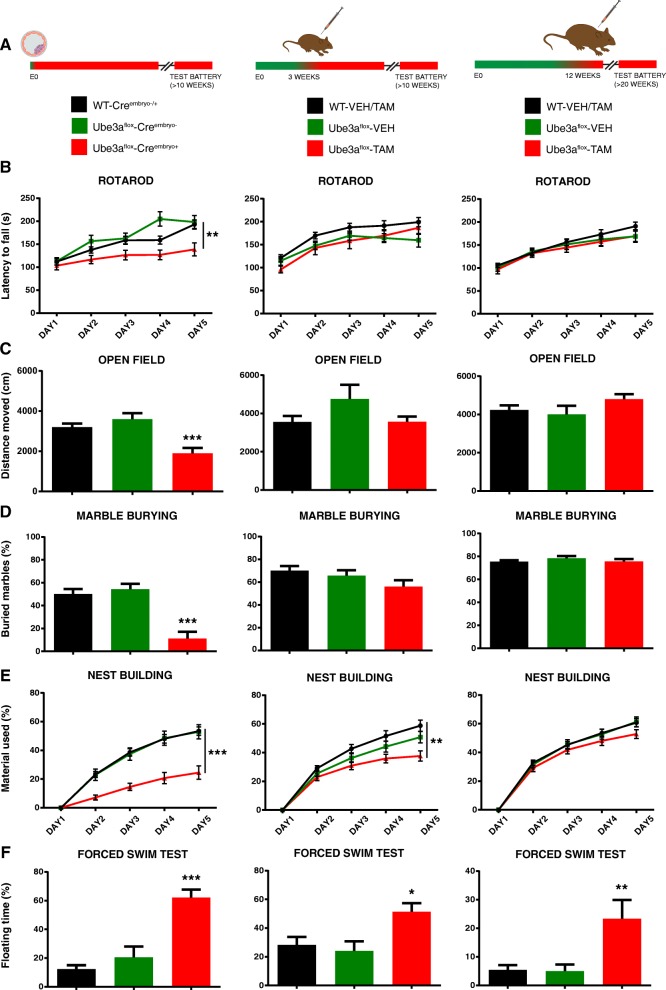
*Ube3a* gene deletion in juvenile and adult mice does not recapitulate the phenotypes observed in embryonically deleted *Ube3a* mice. **a** Schematic depicting *Ube3a* gene deletion at early embryonic age, juvenile age (3 weeks) and adult age (12 weeks). **b**–**f** Behavioral tasks performed with Cre^embryo^;*Ube3a*^*mflox/p+*^ and Cre^ERT^;*Ube3a*^*mflox/p+*^ mice. Juvenile and adult *Ube3a* gene deletion results in deficits in the forced swim test. Asterisks indicate the effect of genotype. Wild-type (WT) mice in the Cre^embryo^;*Ube3a*^*mflox/p*^ group represent combined data of Cre positive and Cre negative animals (embryonic deletion: N for WT-Cre^embryo−^/ WT-Cre^embryo+^ / *Ube3a*^*mflox/p+*^-Cre^embryo^−/ *Ube3a*^*mflox/p+*^-Cre^embryo+^ mice = 15/group). Wild-type mice (WT) in the juvenile and adult-treated gene deletion group represent combined data of tamoxifen and vehicle-treated wild-type mice (Juvenile deletion: N for WT-OIL/ WT-TAM / *Ube3a*^*mflox/p+*^-VEH/ *Ube3a*^*mflox/p+*^-TAM mice = 11, 13, 14, 16) (Adult deletion: N for WT-OIL/ WT-TAM / *Ube3a*^*mflox/p+*^-VEH/ *Ube3a*^*mflox/p+*^-TAM mice = 15/group). Data shown are means with SEM (see methods and Additional file 4 for statistical tests)

To elucidate the importance of continued UBE3A expression after early brain development, we took advantage of a conditional *Ube3a*^*mflox/p+*^ mouse model [20] that enabled us to delete the maternal *Ube3a* gene at any desired time point. We first crossed female *Ube3a*^*mflox/p+*^ mice with a constitutive Cre-expressing mouse line [21]. This resulted in full, early embryonic deletion of the maternal *Ube3a* allele, and a consequent depletion of neuronal UBE3A protein expression in cortex, hippocampus, striatum and cerebellum, similar to what has been observed for the *Ube3a*^*m−/p+*^ AS mouse model [18] (Fig. 1, Additional file 1: Table S1, Additional file 2: Figure S1, Additional file 3: Table S2). Next, we demonstrated that Cre^embryo^;*Ube3a*^*mflox/p+*^ faithfully recapitulate the phenotypes that we previously established to be present in three independent AS mouse models [18, 19] (Fig. 2, Additional file 4: Table S3, Additional file 5: Figure S2, Additional file 6: Table S4). These behavioral phenotypes are in the domains of motor function, anxiety and repetitive behaviors and were selected to fulfill the following criteria: (1) large effect sizes to allow multiple comparisons within and between cohorts, (2) reproducible phenotypes across multiple AS lines, (3) phenotypic penetrance in AS mice of different ages and (4) tolerance of different experimenters. Moreover, these tests can all be performed in a single cohort of mice using a highly standardized (and optimized) method [19].

Having established that *Ube3a*^*mflox/p+*^ mice phenocopy other AS lines tested in our behavioral test battery, we investigated the importance of continued UBE3A expression in these behavioral paradigms, *after* early brain development and into adulthood. To that end, we crossed female *Ube3a*^*mflox/p+*^ mice with a tamoxifen inducible Cre line (Cre^ERT^) [22]. *Ube3a* gene deletion was induced by IP injection of tamoxifen in juvenile mice at 3 weeks of age, and in adult mice at 12 weeks of age (Fig. 2). Western blot analysis of mice sacrificed at approximately 20–25 weeks showed that overall UBE3A protein levels in cortex, hippocampus and striatum of Cre^ERT^;*Ube3a*^*mflox/p+*^ mice closely resembled UBE3A levels of brains in which *Ube3a* was deleted embryonically (Fig. 1; see also Additional file 7: Figure S3, Additional file 8: Figure S4; Additional file 3: Table S2). Immunohistochemistry confirmed tamoxifen-induced *Ube3a* gene deletion throughout the brain (Additional file 7: Figure S3, Additional file 8: Figure S4).

Having ascertained that tamoxifen efficiently deleted the maternal *Ube3a* gene, we assessed the behavioral phenotypes of these Cre^ERT^;*Ube3a*^*mflox/p+*^ mice, minimally 7 weeks after gene deletion. This extended time period between gene deletion and testing not only allowed full clearance of UBE3A protein, but also allowed the neurons and neuronal networks to adapt to the loss of UBE3A expression, ensuring that any observed phenotypes were due to permanent consequences of UBE3A loss.

Maternal *Ube3a* gene deletion in adult (12 week old) mice resulted in impaired forced-swim test behavior. Surprisingly however, performance in the accelerating rotarod, open field, nest building and marble burying paradigms was not affected (Fig. 2; see also Additional file 5: Figure S2). This indicates that these behaviors and their supporting neural circuits do not depend on continued UBE3A expression during adulthood [18]. Importantly, the lack of phenotypic penetrance in these tests is not caused by the age of testing (approximately 20–22 weeks), since we have previously shown that these phenotypes are still clearly present in AS mice aged 28 weeks [18]. Moreover, a retrospective analysis of several studies in our laboratory in which mice aged > 20 weeks were included, showed a strong phenotype on all these tests (Additional file 9: Figure S5).

For experiments in which *Ube3a* gene deletion was induced in juvenile mice at 3 weeks of age, we chose again to delay behavioral testing for a minimum of 7 weeks to allow the brain to respond to the gene deletion. Consequently, the age of testing was similar between juvenile deletion and embryonic deletion mice (Cre^embryo^;*Ube3a*^*mflox/p+*^), allowing for a direct comparison between these groups. Maternal *Ube3a* gene deletion in juvenile mice resulted in a significant impairment in the forced-swim test, highlighting once more the necessity of continued UBE3A expression for normal performance on this test. In addition, these mice also showed an impairment in the nest-building task. Since this phenotype was not present in mice in which *Ube3a* gene deletion was induced at 12 weeks of age, this result indicates that the neuronal network supporting performance in the nest-building task is not yet fully developed at 3 weeks of age. Surprisingly, none of the other tests revealed impairments, suggesting that by 3 weeks of age, the brain has already developed to such an extent that UBE3A protein is no longer required for normal performance of most behaviors.

Most individuals with AS suffer from epilepsy. We have previously shown that AS mice in the 129S2 background exhibit exaggerated susceptibility to audiogenic seizures, which can be suppressed by modulating CAMK2 activity as well as by anti-epileptic drugs [18, 19, 25]. This phenotype is not age dependent as it is readily observed in AS mice tested between 8 and 28 weeks old [18, 19]. We tested sensitivity to audiogenic seizures in 129S2- backcrossed *Ube3a*^*mflox/p+*^ mice in which the *Ube3a* gene was deleted embryonically, at 3 weeks of age or in adulthood. Early embryonic deletion of the *Ube3a* gene rendered all (15/15) Cre^embryo^;*Ube3a*^*mflox/p+*^ mice susceptible to audiogenic seizures. In contrast, neither juvenile (0/8) nor adult *Ube3a* gene deletion (0/16) resulted in mice that were sensitive to audiogenic seizures. This indicates that the sensitivity to audiogenic seizure is exclusively dependent on the presence or absence of UBE3A during early brain development [18].

## Discussion

The purpose of this study was to assess the role of the *Ube3a* gene in the mature brain, with the specific goal of gaining insight into whether UBE3A reinstatement therapies must be sustained throughout life for maximal efficacy in treating AS. In order to address this question, we took advantage of the conditional *Ube3a*^*mflox/p+*^ mouse model [20], crossed with either a constitutive Cre-expressing mouse line (Cre^embryo^) [21] or with a tamoxifen-inducible Cre line (Cre^ERT^) [22], to allow deletion of the *Ube3a* gene at distinct times during brain development.

By deleting *Ube3a* during early embryogenesis, we were able to reproduce all the behavioral deficits observed in various AS mouse models, highlighting the usefulness of this mouse model and the robustness of these phenotypes [18, 19]. These results further confirm the critical role of UBE3A during brain development, as we established previously [18]. In contrast, we observed limited phenotypic penetrance upon *Ube3a* deletion at 3 weeks or 12 weeks of age. We observed no deficits in motor coordination (rotarod), explorative behavior and anxiety (open field), or repetitive behavior and anxiety (marble burying). Nor did we evince a predisposition toward epilepsy as assessed by the audiogenic seizure provocation test. These results cannot be explained by the age of the mice at the time of testing: juvenile deletion mice were the same age at testing as Cre^embryo^;*Ube3a*^*mflox/p+*^ mice in which the gene was deleted embryonically. Moreover, AS mice older than 20 weeks of age continue to exhibit robust phenotypes on these tasks, as we demonstrated both here and in a previous study [18].

Our results corroborate findings from our reciprocal *Ube3a* reinstatement studies [18], leading us to conclude that the circuits underlying these behaviors are brought online during the perinatal period, and are well established by weaning. In contrast, it appears that circuits supporting nest-building behavior are not yet fully mature at 3 weeks of age, since deletion of *Ube3a* at this age (but not at 12 weeks) still results in a significant deficit. Notably, irrespective of the age of the mice, deletion of *Ube3a* always caused a deficit in the forced swim test paradigm, suggesting that the requisite circuits must sustain UBE3A expression for normal performance on this task.

### Limitations

Our study has several limitations. First of all, it is possible that tamoxifen-induced postnatal *Ube3a* gene deletion does not occur in all cells. Our Western blot analysis shows no significant differences between UBE3A protein levels in mice with an embryonic *Ube3a* deletion compared to mice with a postnatal deletion of *Ube3a*. But this does not rule out the possibility that a small percentage of neurons did not undergo *Ube3a* gene deletion following tamoxifen treatment, and that *Ube3a* expression in a small subset of cells is sufficient to maintain normal behavioral function. A second limitation is that we did not assess behaviors related to learning and memory. Individuals with AS show severe intellectual deficits, but as discussed previously [19], AS mouse models do not show robust learning deficits in our hands. Hence, we cannot exclude that normal learning and memory requires UBE3A to be present at a time when learning takes place. Finally, the face validity of some of our behavioral tests is quite limited (e.g. marble burying and nest building), as we do not know the underlying circuits and the relevance of these circuits to human AS phenotypes.

## Conclusions

Our findings underscore the critical role of UBE3A for normal brain development and suggest that most AS behavioral phenotypes arise from the absence of UBE3A during embryonic or early postnatal development. Our results also demonstrate that while expression of UBE3A in the mature brain may not be required for the acquisition and performance of most tests investigated in this study, certain behaviors do depend on continued UBE3A expression. Hence, our study indicates that there is likely to be a clinical benefit by having enduring UBE3A reinstatement. Although we do not know how the first 3 weeks of postnatal brain development in mice translates to human brain development, our results suggest that even transient UBE3A reinstatement during a critical window of early development is likely to prevent most adverse Angelman syndrome phenotypes. Taken together these results emphasize the need to start *Ube3a* gene reactivation therapies early in life, and to sustain reactivation into adulthood for optimal effect.

## Additional files

Additional file 1: **Table S1.** (referring to Fig. 1). Summary of the statistical tests used for each Western blot analysis performed on each experimental group. Statistical significance (2-sided, *p* < 0.05) is indicated by green color. (XLS 31 kb)

Additional file 2: **Figure S1.** Deletion of UBE3A during embryogenesis. A. Whole brain immunohistochemical stainings indicate reduced UBE3A protein levels in *Ube3a*^*flox*^*-Cre*^*embryo+*^ mice compared to *Ube3a*^*flox*^*-Cre*^*embryo–*^ control mice. B. *Ube3a* gene deletion upon CRE activation driven by the Cag promoter during embryogenesis. C. Western blot data indicate reduced UBE3A protein levels in *Ube3a*^*flox*^*-Cre*^*embryo+*^ mice compared to control groups. Number of mice used for the Western blot analysis is *n* = 3 per genotype. Data shown are mean (±SEM). See Additional file 3 (Table S2) for statistical analysis and the sample sizes. (PDF 85 kb)

Additional file 3: **Table S2.** (referring to Additional file 2: Figure S1, Additional file 4: Figure S3, Additional file 8: Figure S4). Summary of the statistical tests used for each Western blot analysis performed on each experimental group. Statistical significance (2-sided, *p* < 0.05) is indicated by green color. (XLS 30 kb)

Additional file 4: **Table S3.** (referring to Fig. 2). Summary of the statistical tests used for behavioral paradigms performed on each experimental group. Statistical significance (2-sided, *p* < 0.05) is indicated by green color. The genotype is the independent variable of all the statistical tests. (XLS 41 kb)

Additional file 5: **Figure S2.** *Ube3a* gene deletion in juvenile and adult mice does not recapitulate the phenotypes observed in embryonically deleted *Ube3a* mice. A. Schematic depicting *Ube3a* gene deletion at early embryonic age, juvenile age (3 weeks) and adult age (12 weeks). B-F. Behavioral tasks performed with Cre^embryo^;*Ube3a*^*mflox/p+*^ (N for WT-Cre^embryo−^/ WT-Cre^embryo+^ / *Ube3a*^*mflox/p+*^-Cre^embryo^−/ *Ube3a*^*mflox/p+*^-Cre^embryo+^ mice = 15/group) and Cre^ERT^;*Ube3a*^*mflox/p+*^ mice (Juvenile deletion: N for WT-OIL/ WT-TAM / *Ube3a*^*mflox/p+*^-VEH/ *Ube3a*^*mflox/p+*^-TAM mice = 11, 13, 14, 16; Adult deletion: N for WT-OIL/ WT-TAM / *Ube3a*^*mflox/p+*^-VEH/ *Ube3a*^*mflox/p+*^-TAM mice = 15/group) . Juvenile and adult *Ube3a* gene deletion results in deficits in the forced swim test. Asterisks indicate the effect of genotype. Data shown are means with SEM. See methods and Additional file 6 for statistical tests and sample sizes. (PDF 401 kb)

Additional file 6: **Table S4. **(referring to Additional file 5: Figure S2). Summary of the statistical tests used for behavioral paradigms performed on each experimental group. Statistical significance (2-sided, *p* < 0.05) is indicated by green color. The genotype is the independent variable of all the statistical tests. (XLS 41 kb)

Additional file 7: **Figure S3.** Deletion of UBE3A in young mice. A. Immunohistochemical staining indicate reduced UBE3A protein levels in *Ube3a*^*flox*^*-*TAM mice compared to *Ube3a*^*flox*^-VEH control group. B. *Ube3a* gene deletion induced at 3 weeks of age upon CRE activation by tamoxifen injection. C. Western blot data indicate reduced UBE3A protein levels in *Ube3a*^*flox*^*-Cre*^*embryo+*^ mice compared to control groups. Number of mice used for the Western blot analysis is *n* = 3–4 per genotype. Data shown are mean (±SEM). See Additional file 3: Table S2 for statistical analysis and the sample sizes. (PDF 1445 kb)

Additional file 8: **Figure S4.** Deletion of UBE3A in adult mice. A. Immunohistochemical stainings indicate reduced protein levels of BE3A in Ube3a^flox^-TAM mice compared to Ube3a^flox^-VEH control group. B. *Ube3a* gene deletion at 12 weeks of age upon CRE activation by tamoxifen injection C. Western blot data indicate reduced UBE3A protein levels in *Ube3a*^*flox*^*-Cre*^*embryo+*^ mice compared to control groups. Number of mice used for the Western blot analysis is *n* = 3/genotype. Data shown are mean (±SEM). See Additional file 3: Table S2 for statistical analysis and the sample sizes. (PDF 51 kb)

Additional file 9: **Figure S5.** Behavioral test battery in mice older than 20 weeks of age. A. Accelerating rotarod in wild-type (WT) and AS mice (*n* = 51, 67). B. Nest building test in WT and AS mice (*n* = 39, 45). C. Open field test in WT and AS mice (*n* = 36, 57). D. Marble burying test in WT and AS mice (*n* = 47, 62). E. Forced swim test in WT and AS mice (*n* = 37, 60). All data represent mean ± SEM. A repeated measures ANOVA or t-test (or Mann Whitney *U* test for nonparametric data) was used for statistical comparison. All tests show a significance effect of genotype (***p < 0.001). (PDF 142 kb)

## Abbreviations

AS: Angelman syndrome
ASD: Autism Spectrum Disorder
ASO: Antisense oligonucleotides
CAMK2: Ca^2+^/calmodulin-dependent protein kinase 2
UBE3A: Ubiquitin-protein ligase E3A
UBE3A-ATS: Ubiquitin-protein ligase E3A antisense transcript
WT: Wild-type

## References

[1] Buiting K, Williams C, Horsthemke B (2016). Angelman syndrome-insights into a rare neurogenetic disorder. Nat Rev Neurol.

[2] Elgersma Y (2015). A molecular tightrope. Nature..

[3] Trillingsgaard A, Østergaard JR (2004). Autism in Angelman syndrome. Autism..

[4] Moss J, Howlin P. Autism spectrum disorders in genetic syndromes: implications for diagnosis, intervention and understanding the wider autism spectrum disorder population. J Intellect Disabil Res. 2009:852–73. 10.1111/j.1365-2788.2009.01197.x 19708861

[5] Peters SU, Horowitz L, Barbieri-Welge R, Taylor JL, Hundley RJ (2012). Longitudinal follow-up of autism spectrum features and sensory behaviors in Angelman syndrome by deletion class. J Child Psychol Psychiatry.

[6] Moreno-De-Luca D, Sanders SJ, Willsey AJ, Mulle JG, Lowe JK, Geschwind DH (2013). Using large clinical data sets to infer pathogenicity for rare copy number variants in autism cohorts. Mol Psychiatry.

[7] Sanders SJ, He X, Willsey AJ, Ercan-Sencicek AG, Samocha KE, Cicek AE (2015). Insights into autism spectrum disorder genomic architecture and biology from 71 risk loci. Neuron..

[8] Cook EH, Lindgren V, Leventhal BL, Courchesne R, Lincoln A, Shulman C (1997). Autism or atypical autism in maternally but not paternally derived proximal 15q duplication. Am J Hum Genet.

[9] Isles AR, Ingason A, Lowther C, Walters J, Gawlick M, Stöber G (2016). Parental origin of interstitial duplications at 15q11.2-q13.3 in schizophrenia and neurodevelopmental disorders. PLoS Genet.

[10] Finucane BM, Lusk L, Arkilo D, Chamberlain S, Devinsky O, Dindot S (1993). 15q duplication syndrome and related disorders. GeneReviews®.

[11] Hogart A, Wu D, LaSalle JM, Schanen NC (2010). The comorbidity of autism with the genomic disorders of chromosome 15q11.2-q13. Neurobiol Dis.

[12] Urraca N, Cleary J, Brewer V, Pivnick EK, McVicar K, Thibert RL (2013). The interstitial duplication 15q11.2-q13 syndrome includes autism, mild facial anomalies and a characteristic EEG signature. Autism Res.

[13] Noor A, Dupuis L, Mittal K, Lionel AC, Marshall CR, Scherer SW (2015). 15q11.2 duplication encompassing only the *UBE3A* gene is associated with developmental delay and neuropsychiatric phenotypes. Hum Mutat.

[14] Yi JJJ, Berrios J, Newbern JMM, Snider WDD, Philpot BDD, Hahn KMM (2015). An autism-linked mutation disables phosphorylation control of UBE3A. Cell..

[15] Meng L, Ward AJ, Chun S, Bennett CF, Beaudet AL, Rigo F (2014). Towards a therapy for Angelman syndrome by targeting a long non-coding RNA. Nature..

[16] Huang H-S, Allen JA, Mabb AM, King IF, Miriyala J, Taylor-Blake B (2011). Topoisomerase inhibitors unsilence the dormant allele of Ube3a in neurons. Nature..

[17] King IF, Yandava CN, Mabb AM, Hsiao JS, Huang H-S, Pearson BL (2013). Topoisomerases facilitate transcription of long genes linked to autism. Nature..

[18] Silva-santos S, Van Woerden GM, Bruinsma CF, Mientjes E, Jolfaei MA, Distel B (2015). Ube3a reinstatement identifies distinct developmental windows in a murine Angelman syndrome model. J Clin Investig.

[19] Sonzogni M, Wallaard I, Santos SS, Kingma J, du Mee D, van Woerden GM (2018). A behavioral test battery for mouse models of Angelman syndrome: a powerful tool for testing drugs and novel Ube3a mutants. Molecular autism.

[20] Judson MC, Wallace ML, Sidorov MS, Burette AC, Gu B, van Woerden GM (2016). GABAergic neuron-specific loss of Ube3a causes Angelman syndrome-like EEG abnormalities and enhances seizure susceptibility. Neuron..

[21] Sakai K, Miyazaki J (1997). A transgenic mouse line that retains Cre recombinase activity in mature oocytes irrespective of thecreTransgene transmission. Biochem Biophys Res Commun.

[22] Hayashi S, McMahon AP (2002). Efficient recombination in diverse tissues by a tamoxifen-inducible form of Cre: a tool for temporally regulated gene activation/inactivation in the mouse. Dev Biol.

[23] Gu B, Carstens KE, Judson MC, Dalton KA, Rougié M, Clark EP (2019). Ube3a reinstatement mitigates epileptogenesis in Angelman syndrome model mice. J Clin Invest.

[24] Intraperitoneal injection of tamoxifen for inducible Cre-driver lines. Available from: https://www.jax.org/research-and-faculty/resources/cre-repository/tamoxifen

[25] Van WGM, Harris KD, Hojjati MR, Gustin RM, Qiu S, Freire RDA (2007). Rescue of neurological deficits in a mouse model for Angelman syndrome by reduction of alphaCaMKII inhibitory phosphorylation. Nat Neurosci.

