# Supplementary figures and images for: Delayed loss of UBE3A reduces the expression of Angelman syndrome-associated phenotypes
Source: Mol Autism. 2019 May 22;10:23. doi: 10.1186/s13229-019-0277-1 (PMC6532248; doi:10.1186/s13229-019-0277-1)

**A**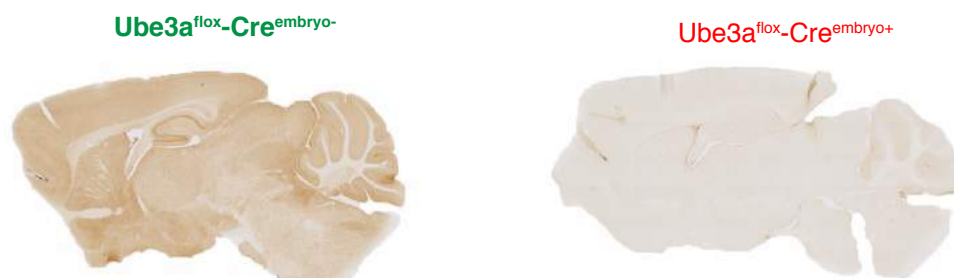**B**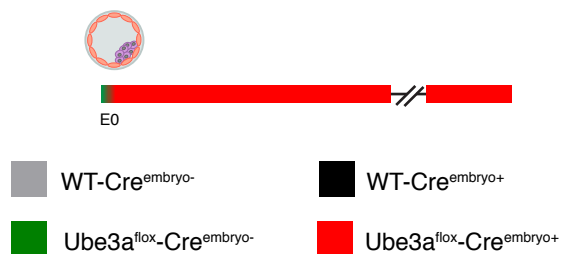**C**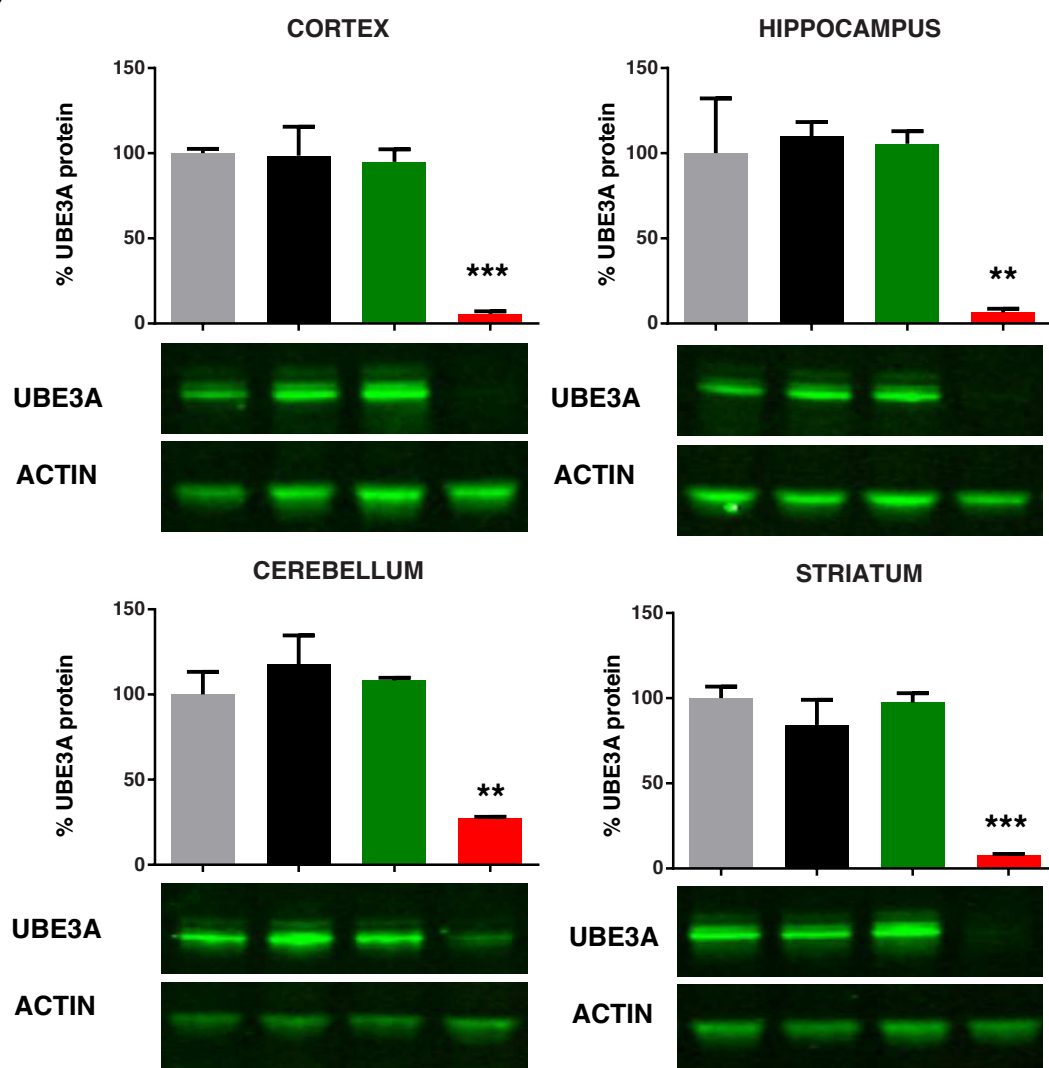

Supplement: Supplementary file 2 — Figure S1. Deletion of UBE3A during embryogenesis. A. Whole brain immunohistochemical stainings indicate reduced UBE3A protein levels in Ube3aflox-Creembryo+ mice compared to Ube3aflox-Creembryo– control mice. B. Ube3a gene deletion upon CRE activation driven by the Cag promoter during embryogenesis. C. Western blot data indicate reduced UBE3A protein levels in Ube3aflox-Creembryo+ mice compared to control groups. Number of mice used for the Western blot analysis is n = 3 per genotype. Data shown are mean (±SEM). See Additional file 3 (Table S2) for statistical analysis and the sample sizes. (PDF 85 kb) [file 13229_2019_277_MOESM2_ESM.pdf]

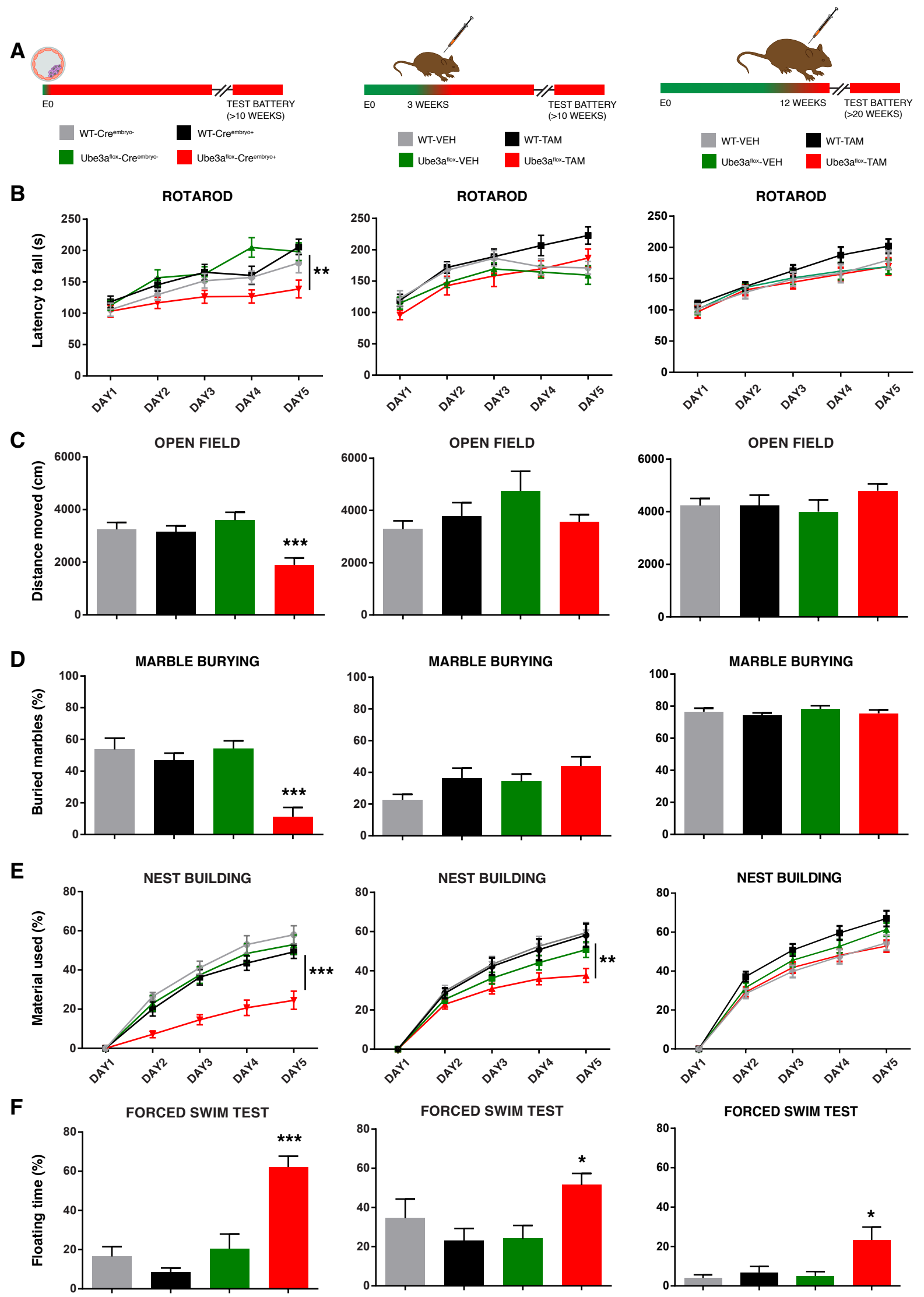

Supplement: Supplementary file 5 — Figure S2. Ube3a gene deletion in juvenile and adult mice does not recapitulate the phenotypes observed in embryonically deleted Ube3a mice. A. Schematic depicting Ube3a gene deletion at early embryonic age, juvenile age (3 weeks) and adult age (12 weeks). B-F. Behavioral tasks performed with Creembryo;Ube3amflox/p+ (N for WT-Creembryo−/ WT-Creembryo+ / Ube3amflox/p+-Creembryo−/ Ube3amflox/p+-Creembryo+ mice = 15/group) and CreERT;Ube3amflox/p+ mice (Juvenile deletion: N for WT-OIL/ WT-TAM / Ube3amflox/p+-VEH/ Ube3amflox/p+-TAM mice = 11, 13, 14, 16; Adult deletion: N for WT-OIL/ WT-TAM / Ube3amflox/p+-VEH/ Ube3amflox/p+-TAM mice = 15/group) . Juvenile and adult Ube3a gene deletion results in deficits in the forced swim test. Asterisks indicate the effect of genotype. Data shown are means with SEM. See methods and Additional file 6 for statistical tests and sample sizes. (PDF 401 kb) [file 13229_2019_277_MOESM5_ESM.pdf]

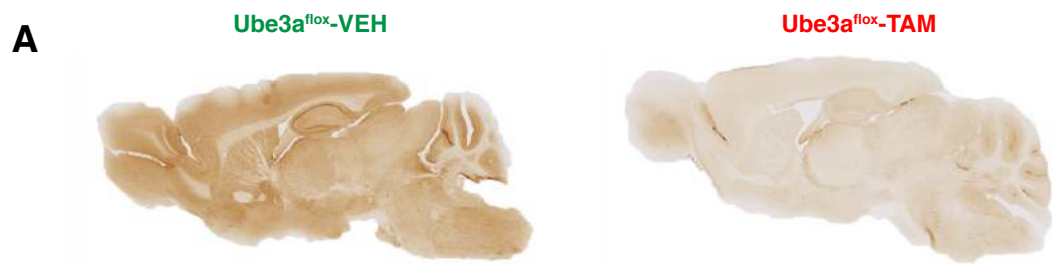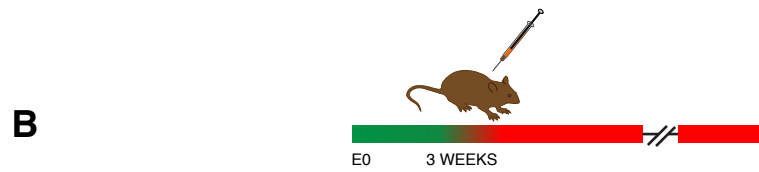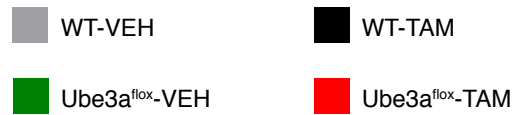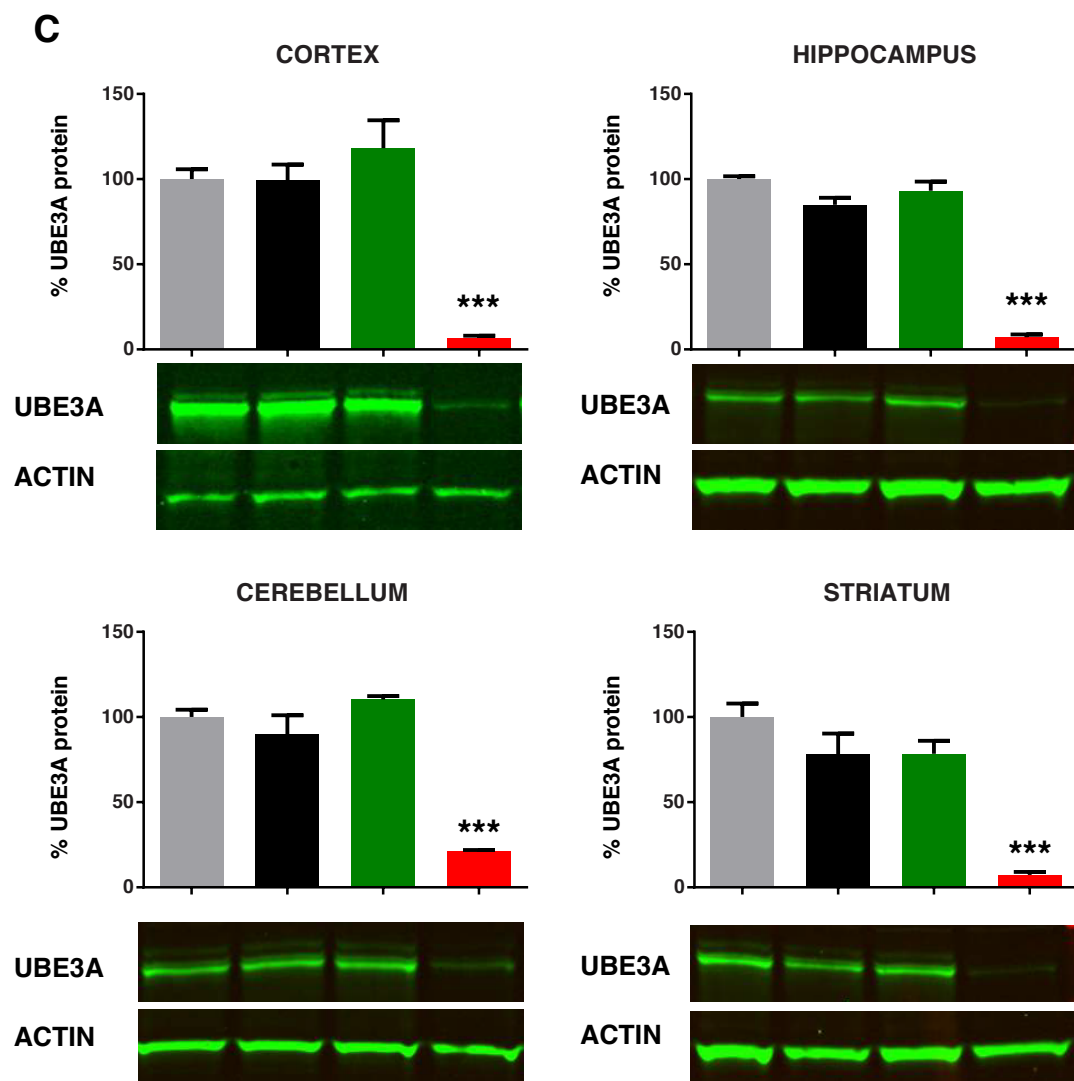

Supplement: Supplementary file 7 — Figure S3. Deletion of UBE3A in young mice. A. Immunohistochemical staining indicate reduced UBE3A protein levels in Ube3aflox-TAM mice compared to Ube3aflox-VEH control group. B. Ube3a gene deletion induced at 3 weeks of age upon CRE activation by tamoxifen injection. C. Western blot data indicate reduced UBE3A protein levels in Ube3aflox-Creembryo+ mice compared to control groups. Number of mice used for the Western blot analysis is n = 3–4 per genotype. Data shown are mean (±SEM). See Additional file 3: Table S2 for statistical analysis and the sample sizes. (PDF 1445 kb) [file 13229_2019_277_MOESM7_ESM.pdf]

**A**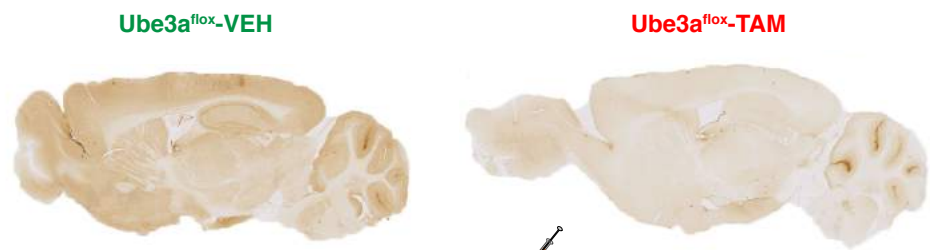**B**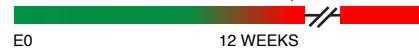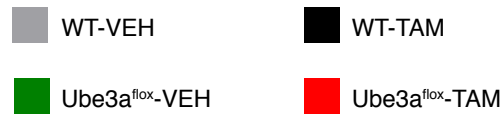**C**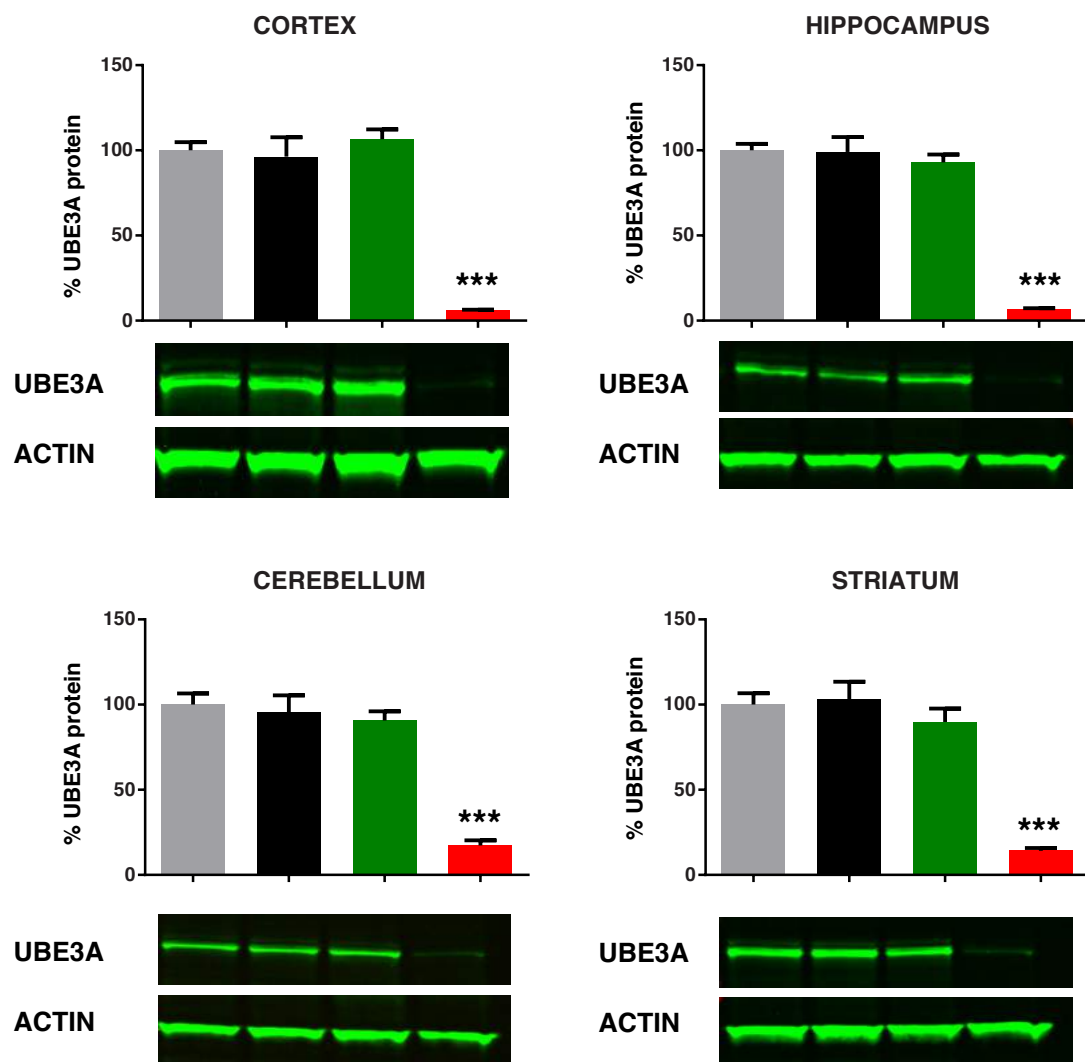

Supplement: Supplementary file 8 — Figure S4. Deletion of UBE3A in adult mice. A. Immunohistochemical stainings indicate reduced protein levels of BE3A in Ube3aflox-TAM mice compared to Ube3aflox-VEH control group. B. Ube3a gene deletion at 12 weeks of age upon CRE activation by tamoxifen injection C. Western blot data indicate reduced UBE3A protein levels in Ube3aflox-Creembryo+ mice compared to control groups. Number of mice used for the Western blot analysis is n = 3/genotype. Data shown are mean (±SEM). See Additional file 3: Table S2 for statistical analysis and the sample sizes. (PDF 51 kb) [file 13229_2019_277_MOESM8_ESM.pdf]

AS  
WT

A

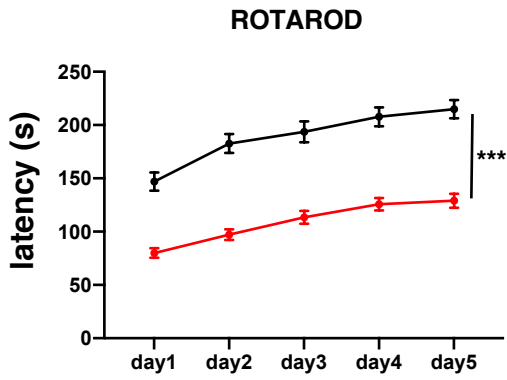

B

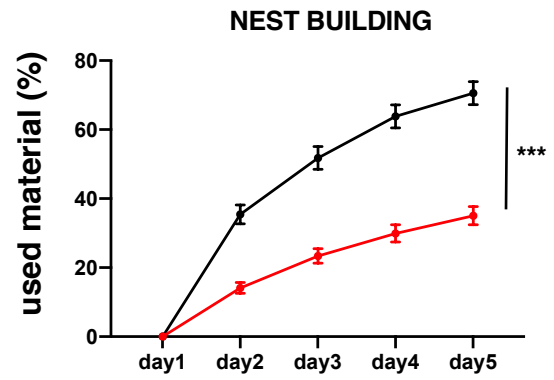

C

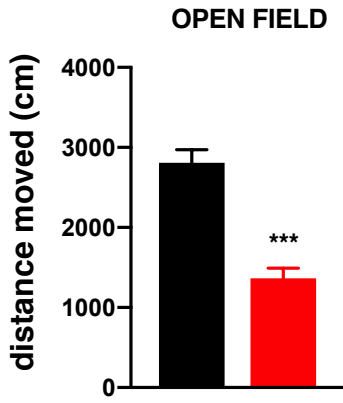

D

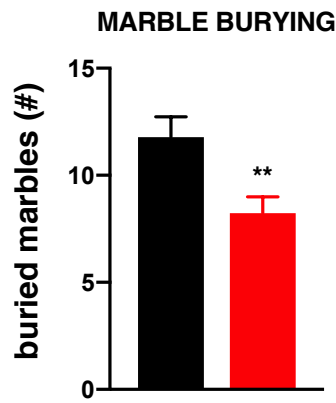

E

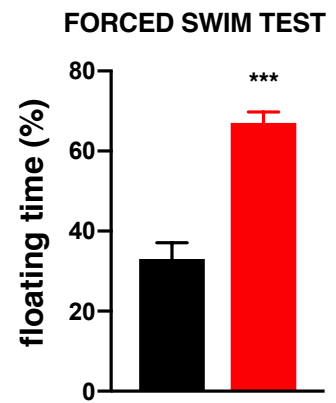

Supplement: Supplementary file 9 — Figure S5. Behavioral test battery in mice older than 20 weeks of age. A. Accelerating rotarod in wild-type (WT) and AS mice (n = 51, 67). B. Nest building test in WT and AS mice (n = 39, 45). C. Open field test in WT and AS mice (n = 36, 57). D. Marble burying test in WT and AS mice (n = 47, 62). E. Forced swim test in WT and AS mice (n = 37, 60). All data represent mean ± SEM. A repeated measures ANOVA or t-test (or Mann Whitney U test for nonparametric data) was used for statistical comparison. All tests show a significance effect of genotype (***p < 0.001). (PDF 142 kb) [file 13229_2019_277_MOESM9_ESM.pdf]
